# Ratiometric electrochemical OR gate assay for NSCLC-derived exosomes

**DOI:** 10.1186/s12951-023-01833-2

**Published:** 2023-03-24

**Authors:** Fanyu Meng, Wenjun Yu, Minjia Niu, Xiaoting Tian, Yayou Miao, Xvelian Li, Yan Zhou, Lifang Ma, Xiao Zhang, Kun Qian, Yongchun Yu, Jiayi Wang, Lin Huang

**Affiliations:** 1 Department of Clinical Laboratory Medicine, Shanghai Chest Hospital, Shanghai Jiao Tong University School of Medicine, Shanghai, 200030 China; 2 Shanghai Institute of Thoracic Oncology, Shanghai Chest Hospital, Shanghai Jiao Tong University School of Medicine, Shanghai, 200030 China; 3 State Key Laboratory for Oncogenes and Related Genes, School of Biomedical Engineering, Institute of Medical Robotics and Med-X Research Institute, Shanghai Jiao Tong University, Shanghai, 200030 China

**Keywords:** Ratiometric biosensor, OR logic gate, Exosomes, Non-small cell lung cancer, Clinical diagnostics

## Abstract

Non-small cell lung cancer (NSCLC) is the most common pathological type of LC and ranks as the leading cause of cancer deaths. Circulating exosomes have emerged as a valuable biomarker for the diagnosis of NSCLC, while the performance of current electrochemical assays for exosome detection is constrained by unsatisfactory sensitivity and specificity. Here we integrated a ratiometric biosensor with an OR logic gate to form an assay for surface protein profiling of exosomes from clinical serum samples. By using the specific aptamers for recognition of clinically validated biomarkers (EpCAM and CEA), the assay enabled ultrasensitive detection of trace levels of NSCLC-derived exosomes in complex serum samples (15.1 particles μL^−1^ within a linear range of 10^2^–10^8^ particles μL^−1^). The assay outperformed the analysis of six serum biomarkers for the accurate diagnosis, staging, and prognosis of NSCLC, displaying a diagnostic sensitivity of 93.3% even at an early stage (Stage I). The assay provides an advanced tool for exosome quantification and facilitates exosome-based liquid biopsies for cancer management in clinics.

**Graphical Abstract:**

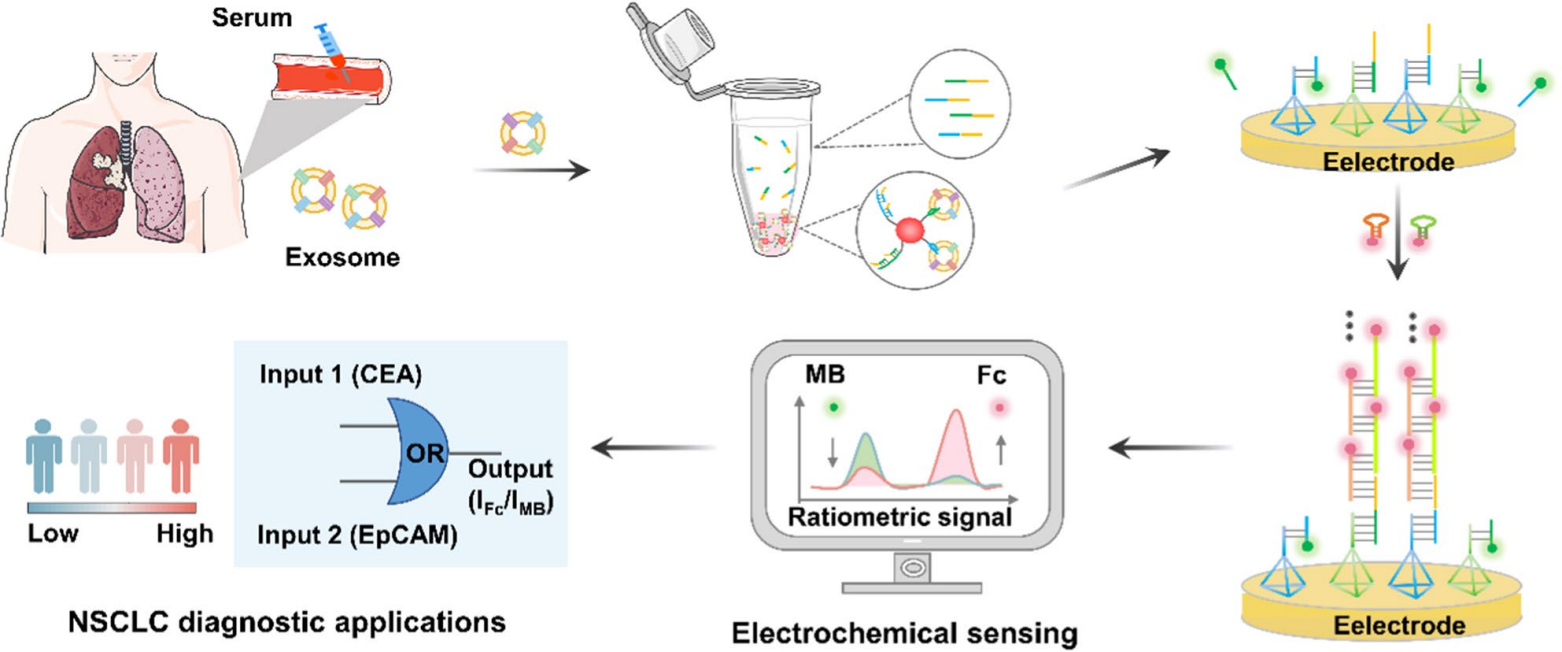

**Supplementary Information:**

The online version contains supplementary material available at 10.1186/s12951-023-01833-2.

## Introduction

Lung cancer (LC) ranks top among all malignancies, leading to a quarter of cancer-related deaths, according to World Health Organization [1]. In particular, non-small-cell lung carcinoma (NSCLC) constitutes about ~ 85% of LC cases, showing a 5-year survival rate as low as 10–15% only [2–4]. Early diagnosis of NSCLC reduces disease mortality with an enhanced five-year survival rate of up to 85%, and precise staging and prognosis relieve disease burden with lowered treatment cost by 61.5% [5, 6]. However, routine methods using tissue biopsy or low-dose computed tomography are limited, concerning nonquantitative examinations, invasive sampling, and poor diagnosis accuracy [7–9]. Therefore, there is an urgent need to develop an assay for clinical diagnosis, staging, and prognosis of NSCLC populations.

Blood test is crucial in liquid biopsy industries and contributes to ~ 66% of clinical diagnosis. In particular, blood contains abundant disease biomarkers, considered information-rich and readily available biospecimens suitable for point of care testing [10–16]. In particular, exosomes are membrane-enclosed nanovesicles (30–150 nm) secreted by diverse cell types into various biological fluids, participating in physiological and pathophysiological processes, *e.g.*, immunomodulation and tumor promotion [17–19]. Exosomes serve as potent mediators of intercellular communication and demonstrate key roles during tumorigenesis. Especially, the NSCLC-derived exosomes from tumor cells (*e.g.*, A549, H460, and H1299) carry different expression levels of multitudinous proteins (*e.g.*, epithelial cell adhesion molecule (EpCAM) and carcinoembryonic antigen (CEA)), resulting in distinct surface phenotypes correlated with cancer occurrence and progression [20–22]. Further considering the high concentration (up to 10^11^ mL^−1^) and stability in circulating blood, surface protein phenotypes of exosomes are explored as a promising biomarker in liquid biopsies for NSCLC patients [23].

Nowadays, most studies regarding exosome surface proteins mainly focus on discovering biomarkers through mass spectrometry or western blots [24]. Little progress has been made in their adaption to the clinical diagnosis of NSCLC, due to the lack of feasible and accurate profiling tools. Electrochemical sensing assays have been developed for the simple detection of disease biomarkers, while suffering from unsatisfactory specificity and sensitivity in exosome profiling [25–27]. For specificity, human exosomes have more heterogeneous compositions than cell line-derived exosomes, and thus it is difficult to obtain comprehensive information of multiple surface proteins on exosomes [28–30]. DNA logic gates are capable of modeling complicated networks and leveraging valuable information within observed data for the accurate estimation and prediction of practical samples [31–33]. They have shown superior performance in analyzing electrochemical signals in complex samples, thus becoming trustworthy solutions to this issue [34, 35]. For sensitivity, the concentration of exosome biomarkers is ultralow at the early stage of NSCLC (*e.g.*, 7 ng/mL) [30, 36]. Unlike the previous assay using electrochemical absolute values of a single reporter, ratiometric methods measure relative signals by two redox reporters (*i.e.*, target-responsive and drift-correcting reference reporters) with opposite variation, offering higher sensitivity with enhanced response magnitude [37]. In addition, ratiometric signals can be further regulated by DNA-based amplification strategies (*e.g.*, hybridization chain reaction (HCR)), to achieve better analytical performance toward trace abundance of targets [38, 39].

**Fig. 1:**
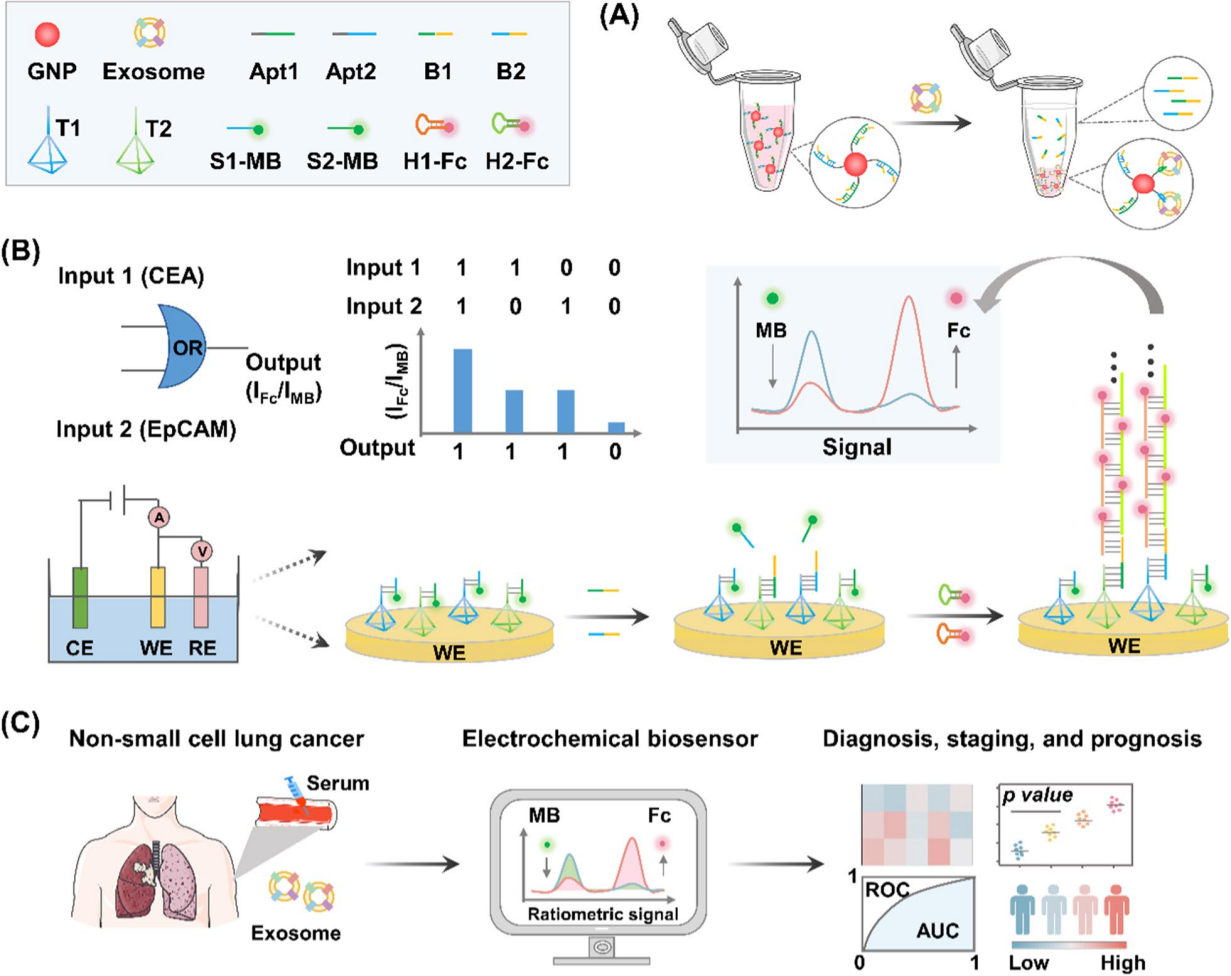
Principle of the ratiometric electrochemical OR gate assay for non-small cell lung cancer (NSCLC)-derived exosomes. **A** Exosomes captured by specific aptamers. **B** Coupling ratiometric electrochemical biosensor with OR logic gate for exosome detection. **C** Workflow of serum exosome detection for the diagnosis, staging, and prognosis of NSCLC cohort. Abbreviations: gold nanoparticle (GNP); Aptamers (Apt1/Apt2); Block strands (B1/B2); DNA tetrahedron (T1/T2); Reference electrode (RE); Counter electrode (CE); Working electrode (WE)

Inspired by the above findings, we presented a ratiometric electrochemical biosensor with the assistance of DNA OR logic gates to form an assay for the detection of multiple NSCLC-derived exosomes (Fig. 1). Two NSCLC-related protein markers, EpCAM and CEA, were selected as targets of exosomes. The DNA probe for OR gate operation contains two parts: an aptamer targeting EpCAM or CEA and an extension region to partially complementary block strands. Upon the recognition of both target proteins on exosome surfaces by OR gate operation probes, block strands were released and separated by the capture of exosome surface proteins via aptamers (Fig. 1A). Then, we acquired the electrochemical readouts as the exosome quantitative signals by targeting to the surface proteins, based on the ratiometric biosensor assisted with DNA OR logic gates and HCR (Fig. 1B). As a proof-of-concept application in clinical diagnostics, we profiled the surface proteins on serum exosomes derived from NSCLC patients and normal controls, suggesting that the differential exosome levels obtained from protein profiling enabling the precise diagnosis, staging, and prognosis of NSCLC (Fig. 1C). Overall, this work establishes a novel liquid biopsy to profile surface proteins and quantify disease-derived exosome in sera, facilitating precision diagnostics of various diseases including but not limited to cancer.

## Experimental methods

### Electrode preparation and electrochemical measurements

Gold electrodes (2 mm diameter) were first pretreated with piranha solution (98% H_2_SO_4_:30% H_2_O_2_ = 3:1) for 5 min (caution: danger, violent reaction). Then, the electrodes were mechanically polished with P4000 silicon carbide paper, and then 1-, 0.3-, and 0.05-mm alumina slurry, respectively. The polished electrode was sonicated in ethanol and ultrapure water for 5 min. Next, the electrodes were electrochemically cleaned with 0.5 M H_2_SO_4_ for 20 cycles to remove any remaining impurities. Finally, the electrodes were dried for mirror-like surfaces, ready to use as working electrodes (WEs). All electrochemical measurements were carried out on a CHI 660E electrochemical workstation (Chenhua Instruments Co., Shanghai, China). The conventional three-electrode system consisted of a DNA-bound gold WE, an Ag/AgCl reference electrode (RE), and a platinum counter electrode (CE). Differential pulse voltammetry (DPV) measurements were performed in 10 mM phosphate buffered saline (PBS, pH 7.4). Electrochemical impedance spectroscopy (EIS) and cyclic voltammetry (CV) measurements were performed in 5 mM [Fe(CN)_6_]^3−/4−^ and 1 M KCl buffer. The experimental parameters were as follows. DPV: scan rate 50 mV/s, sweep range − 0.6–0.7; EIS: bias potential 0.232 V, amplitude 5 mV, frequency range 0.1–100,000 Hz; and CV: sweep range 0.6 to − 0.2 V, scan rate 50 mV/s. The ZSimpWin software was used to fit the Nyquist plots based on a Randles equivalent circuit.

### Aptamer capture of exosome

20 μL of gold nanoparticles (GNPs) were centrifuged at 10,000 rpm for 15 min and resuspended in 1000 μL of ultrapure water for DNA modification. To inactivate aptamer, 1 μM aptamer strand (Apt1/Apt1) and 2 μM block strand (B1/B2) were incubated at 37 ℃ for 1 h to form the Apt1-B1 and Apt2-B2 duplex. Then, a mixture of the DNA duplex (1 μM) at a 1:10 volume ratio was incubated with GNPs at 37 ℃ for 12 h to form a GNPs-DNA structure. The load of oligonucleotides was increased by aging treatment with 2 M NaCl, which was added 6 times every 20 min to a final concentration of 0.1 M. Subsequently, the mixture was centrifuged (10,000 rpm, 10 min) to remove the uncoupled duplex DNA, and the remaining GNPs-DNA conjugates were dispersed in PBS (pH 7.4). Different concentrations of exosomes were added to the solution for 1 h, captured by aptamer strand (Apt1/Apt2) with block strand (B1/B2) released into the solution. After centrifugation, the released block strand (B1/B2) was collected and used for the following experiments. For control experiments, GNPs were modified with one aptamer strand and control strand (C) to form GNPs-DNA structure with Apt1/C or Apt2/C. After the exosome capture, the corresponding released B1 or B2 was collected for further electrochemical detection.

### Fabrication of DNA tetrahedron and exosome detection

DNA tetrahedrons (T1/T2) were assembled by four single-stranded DNAs (D1/D2, D3, D4, and D5) by an annealing process. Oligonucleotide sequences used in the experiments were listed in Supplementary Information (Additional file 1: Table S1). Equimolar amounts of the four DNAs were blended in 20 mM Tris–HCl buffer (50 mM MgCl_2_, pH 8.0). The mixture was heated to 95℃ for 5 min and then slowly cooled down to room temperature for complete hybridization until forming a stable structure. The electrode was modified with 5 μM tetrahedral DNAs (T1/T2, 30 μL) via Au–S bond at room temperature overnight. It was subsequently incubated in 1 mM MCH for 0.5 h to block the nonspecific binding sites, followed by blocking with 5 μM substrate strands (S1-MB/S2-MB) for 1 h. Then, 150 μL of the released block strand (B1/B2), as described above, at various concentrations was incubated with the modified electrode at 37℃ for 1 h and then washed with PBS (10 mM, pH 7.4). Next, 30 μL of a mixture containing 2 μM hairpin DNAs (H1-Fc/H2-Fc) was dropped onto the obtained electrode. The reaction solution was incubated at 37 ℃ for 1.5 h before the electrochemical measurements. The control experiments were prepared separately and performed under the same conditions. All experiments were repeated three times.

Polyacrylamide gel electrophoresis (PAGE) analysis was performed to characterize the DNA tetrahedron fabrication. For DNA tetrahedron characterization, 10 μL of sample 1 (5 μM, D1), sample 2 (5 μM, D2), sample 3 (5 μM, D1 + D3), sample 4 (5 μM, D1 + D3 + D4), sample 5 (5 μM, D1 + D3 + D4 + D5), and sample 6 (5 μM, D2 + D3 + D4 + D5) were placed on a 12% polyacrylamide gel. The electrophoresis was performed in 0.5 × Tris-borate-EDTA (pH 8.0) at 100 V constant voltage for 1.5 h. After that, the gel was scanned using a gel imaging analyzer.

### Study population and serum harvesting

A total of 135 subjects were recruited in Shanghai Chest Hospital, affiliated to Shanghai Jiao Tong University School of Medicine, including 105 NSCLC patients and 30 normal controls (healthy donors (HD, *n* = 15), pneumonia (*n* = 5), bronchitis (*n* = 5), fibrosis (*n* = 5)). All cancerous subjects were verified with pathological results. The tumour was staged according to the international standards for tumour, node, and metastasis (TNM) staging, including 30 stage I, 25 stage II, 25 stage III, and 25 stage IV [40, 41]. NSCLC patients included three cancer types (adenocarcinoma, ADC; squamous-cell carcinoma, SCC; and large-cell carcinoma, LCC). All samples were anonymized, and relevant pathological diagnoses were recorded (Additional file 1: Tables S3–S5).

All blood samples were drawn into BD Vacutainer^®^ SST^™^ Tubes by venipuncture and clotted at room temperature within 40 min. Serum was collected at 3000 × g for 10 min of centrifugation from the blood and immediately stored at – 80 ℃ for further analysis. All the investigation protocols were approved by the Institutional Ethics Committees of Shanghai Chest Hospital, under the approved protocol No. KS22025. All subjects provided informed consent to participate in the study and approved the use of their biological samples for analysis.

### Exosome isolation for clinical assay

For cell model investigation, NSCLC cell lines (A549, H460, H1299, H1975, H2030, and Calu-1 cells) were cultured in Dulbecco's modified Eagle's medium (Gibco, USA) supplemented with 10% fetal bovine serum (Gibco) in 5% CO_2_ in an incubator at 37 °C. At the exponential growth phase, cells were collected with trypsinization and centrifuged at 1000 rpm for 5 min. Exosomes were extracted from the cancer cells using a miRCURY^®^ Exosome Kits (Qiagen, Hilden, Germany). The samples were counted and characterized by NanoFCM Flow NanoAnalyzer (NanoFCM Inc., Xiamen, China), and then stepwise diluted into solutions to different final concentrations.

For clinical serum applications, the exosomes from the serum of HDs, benign diseases, and NSCLC patients were extracted using an ExoQuick^™^ Kit (System Biosciences, USA). Extracted exosomes were firstly spiked into the provided buffer by 100-fold dilutions to different concentrations, for better assessment of the practical utility of the assay.

### Statistical analysis

All statistical analyses in this work (including significance analyses, receiver operating characteristic curve (ROC) construction, and area under curve (AUC) calculation) were performed on IBM SPSS Statistics software (Version 26.0.0). Sensitivity referred to the probability that the assay result indicated "positive" among all cancerous subjects. Specificity was the fraction of those without cancer, which showed a negative assay result. The 95% confidence intervals (CIs) were calculated using a binomial distribution based on receiver operator characteristic analysis. The significant difference was calculated using a two-tailed Student's t-test, with all significance levels set as 5%. Three independent experiments were performed with data shown as the mean ± SD (*n* = 3). Plots and charts were performed using Origin 2021 software.

## Results and discussion

### Principle for exosome detection

 Figure 1 illustrates the assay principle to quantify NSCLC-derived exosomes as a function of surface biomarker expression. CEA and EpCAM, dual biomarkers enriched on the surface of cancer exosomes, were selected as specific targets to achieve this goal [42]. The strategy consists of three steps: (1) construction of DNA OR logic gate for signal input: exosomes competitively bind to CEA and EpCAM aptamers (Apt1/Apt2) and release block strands (B1/B2) from the aptamer-block hybridization complex. (2) Triggering of DNA tetrahedron (T1/T2) for signal output and introduction of HCR for signal amplification: on the electrode surface, the released block strands (B1/B2) hybridize with the primer strands (D1/D2) of the DNA tetrahedron (T1/T2) and displace the substrate strand (S1-MB/S2-MB), generating the decrease of MB intensity (I_MB_). Then, block strands activate HCR with hairpin DNAs (H1-Fc/H2-Fc) to produce long double-stranded DNAs, resulting in a significant signal enhancement of Fc (I_Fc_). Finally, the ratiometric intensity of Fc and MB signal (I_Fc_/I_MB_) is used as signal output, corresponding to the level of exosome. (3) Analysis of serum exosome for diagnostic application: exosomes were detected from serum samples of NSCLC patients and correlated to different disease status, including NSCLC patients with stage I-IV, BDs, and HDs.

For signal input, DNA OR logic gate was designed for multiple exosome biomarkers (CEA and EpCAM) in parallel, to obtain comprehensive tumor information toward precision medicine. In detail, gold nanoparticles (GNPs) were functionalized with particular aptamers (Apt1/Apt2) targeting CEA and EpCAM of exosome to form a DNA OR logic gate. Notably, block strands consist of two parts: one is partially complementary with the aptamer sequence and another is complementary to the primer strands of the DNA tetrahedron. The designed aptamers (Apt1/Apt2) had a higher affinity with exosomes compared with the block strands, leading to the release of block strands (B1/B2) from the aptamer-block hybridization complex. Therefore, the exosome content was converted to the level of released B1/B2.

For signal output, DNA tetrahedron with controlled shape was functionalized on electrode surface to detect the specific binding of signal strands, avoiding steric hindrance and molecular entanglement. In particular, the working electrode (WE) was incubated with substrate strands (S1-MB/S2-MB), in which MB was introduced as reference signal. Once B1/B2 strands hybridize with the primer strands and replace the substrate strands, the MB signal will decrease. As a result, the decrement extent of the reference signal (I_MB_) can serve as an indicating readout for exosome contents. During the test process, the existence of either one single biomarker from exosome surface or two biomarkers led to an electrochemical signal (I_Fc_/I_MB_) output. Only when there is no existence of any biomarker, the DNA OR logic gate provide no electrochemical signal output.

For signal amplification, the trace abundance of recognition biomarkers on exosome surface places obstacle for direct detection [43]. Therefore, we designed a cascade amplification via HCR for the enhancement of Fc signal (I_Fc_). It is worth noting that the intensity of Fc is accompanied by a signal decline of referenced MB, further amplifying the ratiometric current (I_Fc_/I_MB_) for accurate detection of trace exosomes. Specifically, the clinical application of the assay is not limited to LC diagnosis. In another diagnostic scenario targeting different exosome biomarkers (*e.g.*, HER2 for breast cancer), the operation is facile by simply changing the aptamer sequences and the corresponding block strands. Therefore, universal disease diagnosis can be realized through the substitution of multiple aptamers in the assay.

### Characterization of exosome, GNP, and DNA tetrahedron

**Fig. 2:**
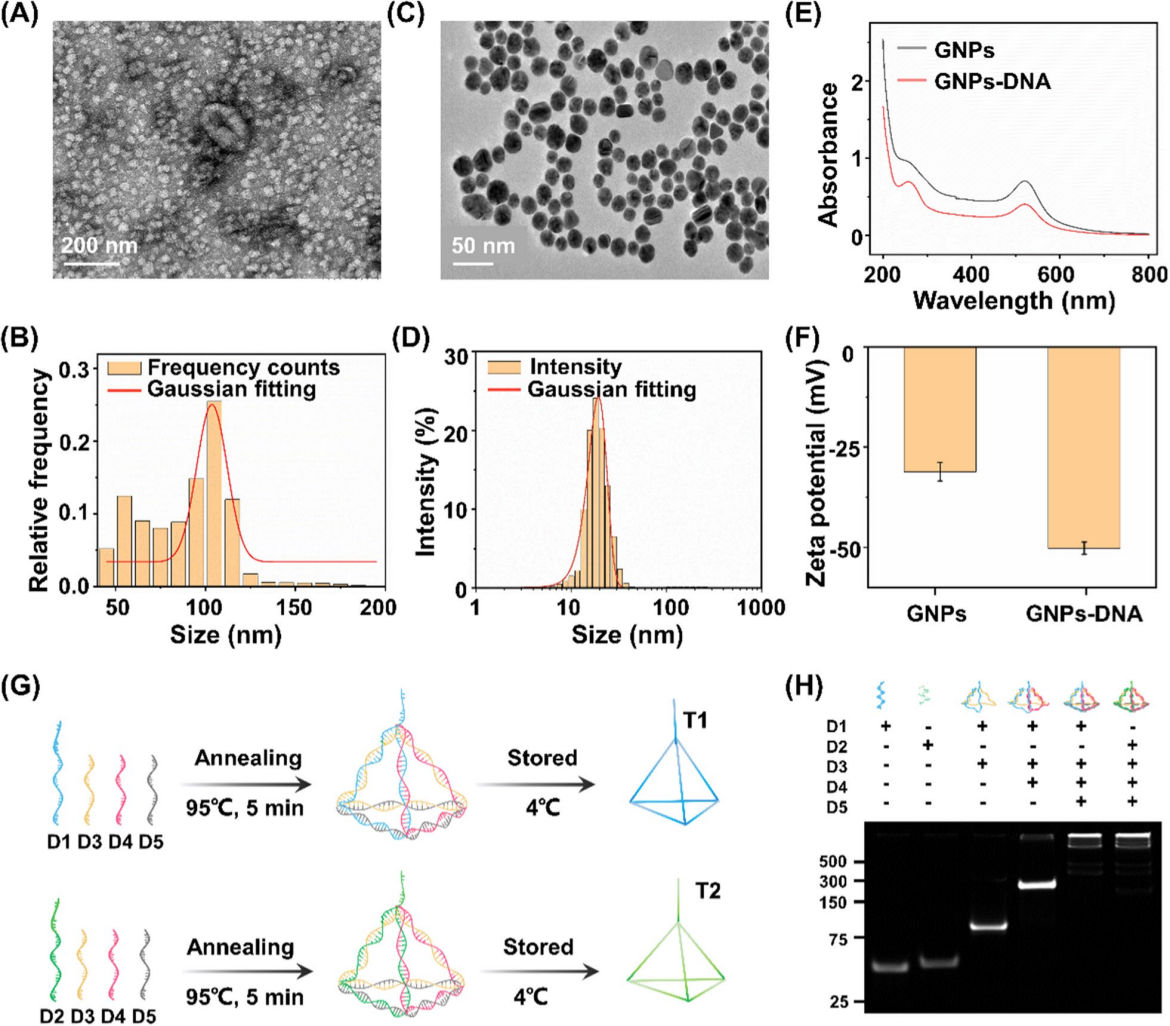
Characterization of exosome, GNP, and DNA tetrahedron. **A** Transmission electron microscopic (TEM) image and **B** nanoparticle flow cytometry (NanoFCM) characterization of exosomes. **C** TEM image and **D** dynamic light scattering analysis of gold nanoparticles (GNPs) dispersed in water at room temperature. **E** Ultraviolet–visible spectroscopy absorption spectra and **F** Zata potential results of the GNPs and aptamer-functionalized GNPs (GNPs-DNA). **G** Assembly process of DNA tetrahedrons (T1/T2). **H** Polyacrylamide gel electrophoresis analysis of T1/T2 (formed by D1/D2, D3, D4 and D5)

By using a commercial extraction kit, exosomes with sizes ranging from 30 to 150 nm were isolated from both NSCLC cell culture media and clinical serum biospecimens of HDs and patients with NSCLC. A flatted round shape was observed in the transmission electron microscopy (TEM) images of exosomes (Fig. 2A). We also examined size parameters of the exosomes extracted from A549 cells by nanoflow cytometry approach. The acquired morphology revealed an average size of ∼100 nm of exosomes (Fig. 2B), consistent with the TEM result as well as previous literature confirming the successful extraction of exosomes [44].

GNPs are critical in the aptamer recognition and capture process (see Methods for details). As shown in TEM image, well-distributed spheres were observed possessing an average diameter of 13 ± 3 nm (Fig. 2C). In the dynamic light scattering experiment, GNPs displayed an average hydrodynamic size of 18 ± 3 nm (Fig. 2D) agreed to TEM characterizations, demonstrating a typical particle size in use according to literature [45]. In a typical ultraviolet–visible absorption spectrum, the light absorption band of GNPs occurred at 520 nm. The new absorption peak at 260 nm, a characteristic band of nucleic acid composites, verified the satisfied functionalization of GNPs with thiol-aptamers (Fig. 2E), in regards to the binding affinity between thiol groups and gold [46]. In addition, GNPs were negatively charged with zeta potential of 31 ± 3 mV (Fig. 2F), beneficial for the distribution of aptamers and preventing non-specific bindings. A significant decrease in zeta potential of GNPs to 50 ± 2 mV (*p* < 0.05) also confirmed the surface modification via positively charged aptamers.

DNA tetrahedrons are the basic structure for the electrochemical reaction, assembled from four single-stranded oligonucleotides (D1/D2, D3, D4, and D5 for T1/T2, respectively) by an annealing process (Fig. 2G). The self-assembly process of DNA tetrahedron was investigated by native polyacrylamide gel electrophoresis (PAGE, Fig. 2H). The lanes from left to right (corresponding to Lane 1 to 5/6) in PAGE results indicated a stepwise increase in molecular weight during self-assembly process. As shown in Lane 5 and 6, the DNA tetrahedrons were prepared through consecutive hybridization among DNA oligonucleotides. In particular, the consecutive bands were witnessed due to decreased mobility, consistent with the significantly increased hydrodynamic sizes of DNA structures. Therefore, the increase in molecular weight as well as mobility together validated successful self-assembly of DNA tetrahedrons on WE.

### Construction and feasibility verification of the biosensor

**Fig. 3:**
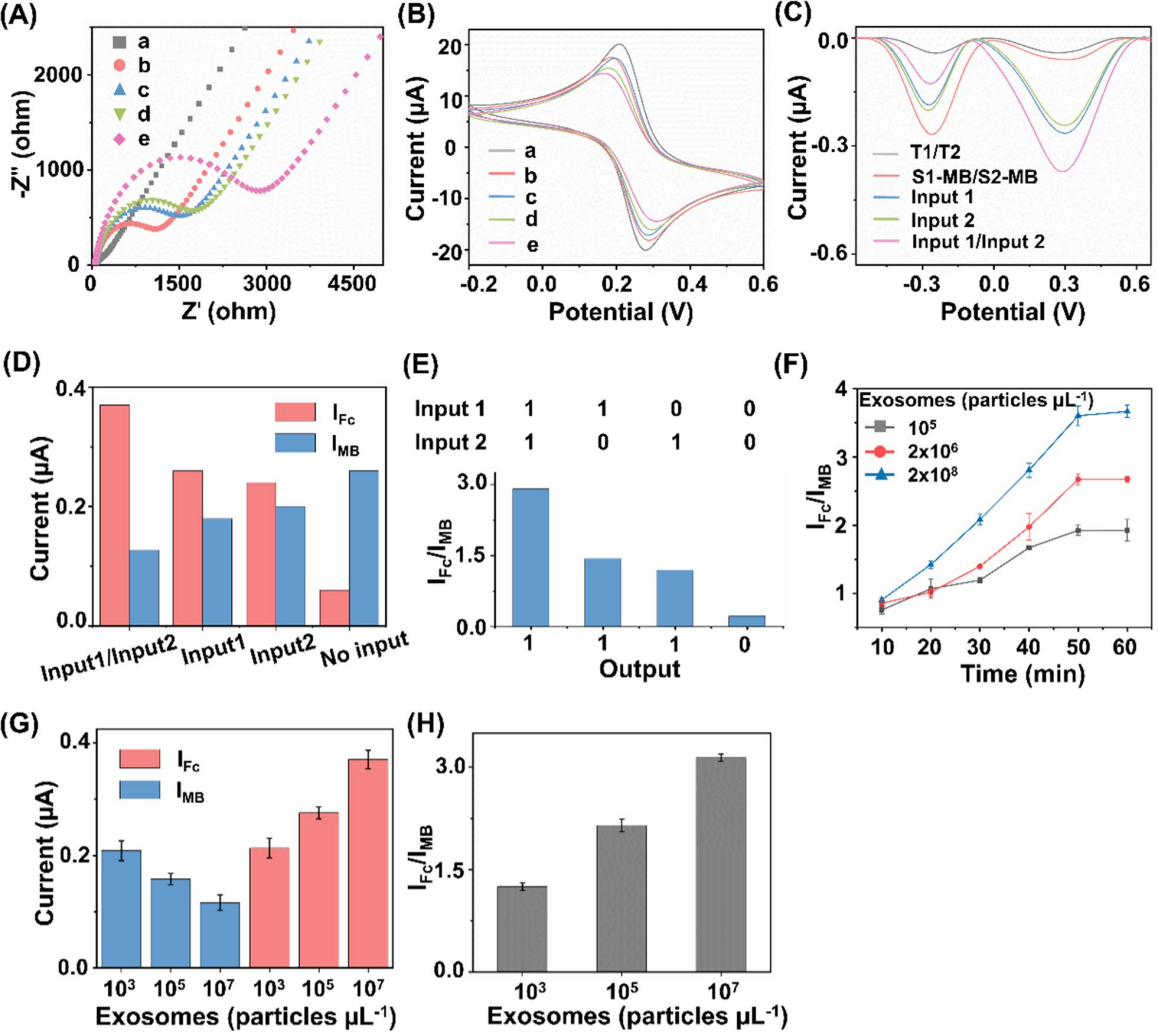
Construction and feasibility verification of the biosensor. **A** Electrochemical impedance spectroscopy and **B** cyclic voltammetry curves of the bare electrode (curve a), bare + T1/T2 (curve b), bare + T1/T2 + S1-MB/S2-MB (curve c), bare + T1/T2 + S1-MB/S2-MB + B1/B2 (curve d), and bare + T1/T2 + S1-MB/S2-MB + B1/B2 + H1-Fc/H2-Fc (curve e). **C** Differential pulse voltammetry (DPV) curves under different experimental conditions. **D** DPV peak current of I_MB_ and I_Fc_ under different signal inputs. **E** Validation of OR logic gate in the presence/absence of epithelial cell adhesion molecule (EpCAM) and carcinoembryonic antigen (CEA). **F** Optimization of incubation time for exosome captured by aptamers. The concentrations of exosomes were set as 10^5^, 2 × 10^6^, and 2 × 10^8^ particles µL^−1^, respectively. **G** Single MB signal (I_MB_), single Fc signal (I_Fc_), and **H **I_Fc_/I_MB_ ratio signal for exosome detection. The concentrations of exosomes were set as 10^3^, 10^5^, and 10^7^ particles µL^−1^, respectively

The stepwise construction process of the biosensor has been validated by electrochemical impedance spectroscopy (EIS) and cyclic voltammetry (CV). As depicted in Fig. 3A, the bare electrode presented an electron transfer resistance (R_et_) of about 72 Ω (curve a). After the successive assembly of T1/T2 and MCH on the electrode, the R_et_ increased significantly (curve b) due to the hindered electron transfer after the conjugation of DNA tetrahedrons. When S1-MB/S2-MB were attached to the electrode surface, the R_et_ value increased further (curve c), which could be ascribed to the repulsion of [Fe(CN)_6_]^3−/4−^ by the DNA backbone. The R_et_ value continued to increase after the replacement of S1-MB/S2-MB by the released B1/B2 after hybridizing B1/B2 with T1/T2 (curve d). In the presence of H1 and H2, the R_et_ value increased considerably (curve e), which implied that the HCR amplification response was initiated on the electrode. Sequential fabrication steps for the ratiometric biosensor were also investigated using CV measurements of the current changes. As seen in Fig. 3B, the voltammograms of [Fe(CN)_6_]^3−/4−^ gradually changed during the modification of the electrodes. All the electrochemical performances indicated the successful fabrication of the biosensor, promising the simultaneous analysis of multiple exosome surface proteins [47, 48].

The feasibility of the electrochemical biosensor for exosome detection was also evaluated. As shown in Fig. 3C, after modified with DNA tetrahedron (T1/T2), barely any signal was observed in the absence of the released B1/B2, S1-MB/S2-MB, and H1-Fc/H2-Fc, indicating that the majority of the signals were not immobilized on the electrode. Once the reference signal from S1-MB/S2-MB was present in the reaction system, we observed an obvious signal at − 0.267 V, indicating the intercalation of MB on electrode surface via hybridization. When the exosome surface protein of CEA or EpCAM was captured by Apt1 or Apt2, the corresponding block strand (B1 or B2) was released into electrolyte and thus served as signal input (Input 1 or Input 2, respectively) for downstream sensing. In the co-existence of CEA and EpCAM, B1 and B2 were released in parallel and served as Input 1/Input 2. In the presence of released block strands (B1, B2, and B1/B2) and H1-Fc/H2-Fc, the electrochemical signal showed a further enhancement of Fc signal at + 0.278 V and a reduced MB signal at − 0.267 V. As a result, the corresponding signal intensities of Fc signal (I_Fc_) and MB signal (I_MB_) were illustrated under different input conditions (Fig. 3D, E). Notably, the current ratio of I_Fc_/I_MB_ (used as "Output") was significantly increased, owing to two signals varied in an opposite manner induced by target exosomes. Therefore, the constructed biosensor is capable of built-in correction analysis, improving sensing sensitivity with DNA-based HCR amplification [49, 50].

We further set out to identify the optimized experimental conditions, including the aptamer DNA volume ratio and the recognition time for exosome capture, as well as the DNA tetrahedron concentrations and HCR amplification time for electrochemical exosome detection. Different volume ratios were first investigated with constant exosomes, and the DPV intensity reached the highest at a concentration of 1:10 (Additional file 1: Fig. S1A). Next, the recognition time between the exosome analytes and aptamers was optimized to 1 h, yielding the highest I_Fc_/I_MB_ for the specific recognition and capture (Fig. 3F). Both T1/T2 concentration and HCR amplification time decide the signal intensity of the electrochemical exosome detection. The value of I_Fc_/I_MB_ increased with the rising T1/T2 concentration, and reached the platform after 5 μM added. As a result, the optimum concentration and amplification time were determined to be 5 μM and 1.5 h respectively (Additional file 1: Fig. S1B, C), offering the saturation of signal binding and improving the amplification efficiency.

Electrochemical sensing is typically designed for single biomarkers, hardly depicting a comprehensive picture under pathological stimuli [51, 52]. To address the analyte limitations, introducing multiple electrochemical active substances or nanostructures are normally required [53–55]. However, lack of sensibility and susceptible to environmental conditions hampers the universal application of the above electrochemical techniques in clinics. Ratiometric biosensors serve as a practical alternative for detection of multiple biomarkers, due to the ability to amplify the signal changes and eliminate the fluctuations by external factors [38, 39, 56, 57]. In this work, single signal biosensors displayed a maximum DPV response within 30% only (current change of 29.4% for I_MB_ and 20.5% for I_Fc_, Fig. 3G). In comparison, our constructed ratiometric biosensor demonstrated a decrease in I_MB_ and an increase in I_Fc_ (Fig. 3H), facilitating the sensitive detection with an increased change of 58.9% in signal ratio (I_Fc_/I_MB_). In addition, the data acquired from ratiometric biosensor has higher reproducibility and accuracy with a lower coefficient of variation (CV) of 3.5% (Fig. 3H), as compared with that from single signal biosensors (CV = 8.9% for I_MB_, CV = 5.5% for I_Fc_; Fig. 3G). Therefore, our approach addressed the current challenges with enhanced biomarker throughput (CEA and EpCAM as a proof-of-concept demonstration in this work) and robust analyte quantitation (lower CV = 3.5%), by introducing a reference signal to construct a ratiometric biosensor.

### Ultrasensitive detection of exosome

**Fig. 4:**
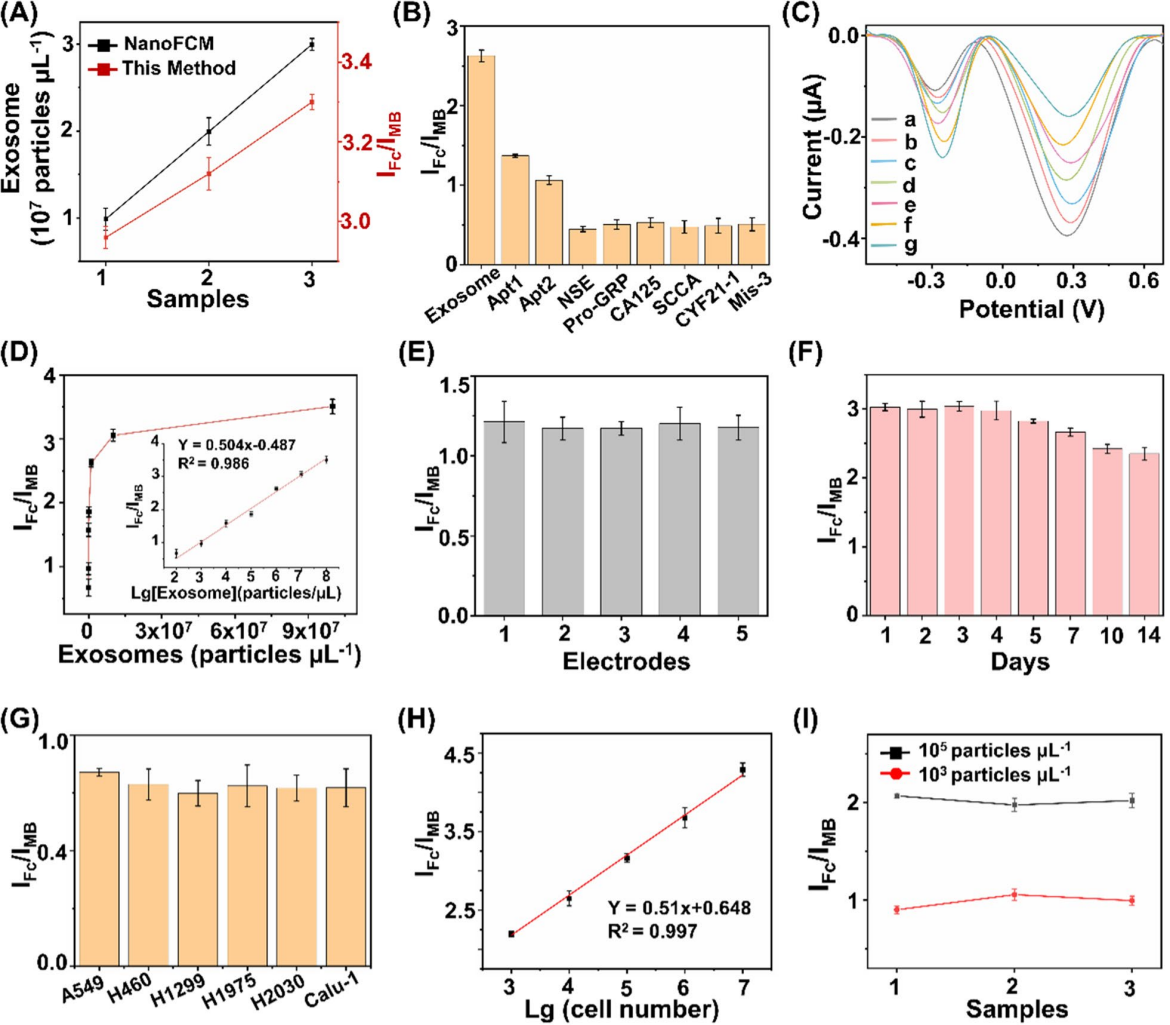
Ultrasensitive detections of exosomes. **A** Comparison of the biosensor with NanoFCM for exosome detection. **B** Determination of the selectivity of exosome detection against typical interfering substances, including 1 KU/mL of CA125, 1 mg/mL of NSE, Pro-GRP, SCCA, and CYF21-1, respectively. **C** DPV analysis of different concentrations of exosomes ranging 10^8^–10^2^ particles µL^−1^ (from a to g). **D** Linear calibration curve between the ratiometric intensities and logarithm of exosome concentrations. **E** DPV responses acquired from 5 working electrodes, under the constant level of exosomes (2 × 10^3^ particles µL^−1^). **F** DPV responses acquired over two weeks, under the constant level of exosomes (10^7^ particles µL^−1^). **G** Exosome expression were profiled in different types of NSCLC cell lines (A549, H460, H1299, H1975, H2030, and Calu-1 cells). **H** Linear correlation between ratiometric intensities I_Fc_/I_MB_ and A549 cell numbers ranging 10^3^–10^7^. **I** Ratiometric intensities of exosomes spiked into three clinical serum samples at final concentrations of 10^3^/10^5^ particles µL^−1^. Error bars referred to the standard derivation obtained from three independent experiments

The proposed ratiometric biosensor enabled the exosome detection in an accurate, specific, sensitive, reproducible, and stable manner. For detection accuracy, we found that quantitation of exosome concentrations using the proposed biosensor were in agreement with those measured via a commonly-used nanoflow cytometry method, with a Pearson correlation coefficient of 0.917 (Fig. 4A). For detection specificity, the assay displayed preference toward exosomes with highest I_Fc_/I_MB_ response (Fig. 4B), against typical interfering substances that co-existed in human blood (*e.g.*, neuron-specific enolase (NSE), squamous cell carcinoma antigen (SCCA), pro-gastrin-releasing peptide (Pro-GRP), carcinoembryonic antigen 125 (CA125), section  19 of CYFRA 21-1 (CYF21-1), and three-base mismatched aptamers (Mis-3)). For detection sensitivity, the subtle changes of different exosomes were determined by the proposed biosensor under optimal experimental conditions (Fig. 4C). The current intensity ratio I_Fc_/I_MB_ was linearly proportional (R^2^ = 0.986) to the exosome concentrations (Fig. 4D), yielding a low limit of detection of 15.1 particles μL^−1^ within a wide linear range of 10^2^–10^8^ particles μL^−1^ (I_Fc_/I_MB_ = 0.504 Lg*C*_exosome_ − 0.487). For detection reproducibility, we recorded current intensities of 2 × 10^3^ particles μL^−1^ of exosomes at intra-batch level (5 parallel measurements for three independent experiments), affording coefficients of variation within 4.8% (Fig. 4E). For detection stability, the electrochemical signals were retained at 88.1% after storage for one week and 77.8% for two weeks (Fig. 4F), indicating the long-term stability suitable for clinical use.

Having optimized the biosensor, we validated the feasibility of our approach in exosome sensing in both cell media and real-case biospecimens. We first profiled the exosome expression in different NSCLC cell lines (A549, H460, H1299, H1975, H2030, and Calu-1 cells). A significant increase in ratiometric intensity was detected in NSCLC cell lines as compared with that in phosphate buffered saline (Fig. 4G), suggesting a positive correlation between the expression levels of surface proteins CEA and EpCAM on the exosomes and LC pathogenesis [30, 36, 42]. Next, we selected A549 cells as an example to validate the capability of the proposed biosensor for exosome quantitation. The peak current difference exhibited a linear correlation with the logarithmic number of cells, yielding a wide linear range of 10^3^–10^7^ cells (Fig. 4H). Exosomes were spiked into human serum to concentrations of 10^3^/10^5^ particles μL^−1^. The biosensor afforded the average recovery of 90.1%-105.6% with CV within 5.4% (Fig. 4I), comparable to the traditional electrochemical method according to previous reports [20].

The analytical performances of the developed biosensor and previously reported methods for exosome detection are summarized in Supplementary Information (Additional file 1: Table S2). As can be seen, the proposed biosensor exhibited a lower limit-of-detection within a wider linear range, compared with other techniques (*e.g.*, fluorescence, surface plasmon resonance, and electrochemiluminescence) [58–60]. We attributed the superior sensitivity of the proposed biosensor to the ratiometric strategy and aptamer-facilitated HCR for signal amplification [61, 62]. In addition to the sensitivity performance, the proposed biosensor also displayed an improved specificity by introducing a DNA OR logic gate for the simultaneous detection of two surface protein targets, which provides more comprehensive information for cancer diagnosis [63–65]. In particular, single biomarker hardly distinguishes patient groups with satisfied performance, since diseases are accompanied by abnormal regulation of multiple biomarkers. The multiplex and simultaneous analysis of multiple biomarkers depicted the systematic alteration under disease stimuli, playing a key role especially in the era of precision medicine. Notably, the distinct signal intensities from different systems suggested a positive correlation between exosome expression and LC pathogenesis (Fig. 4G). Therefore, we concluded that the ratiometric biosensor achieved accurate, specific, sensitive, reproducible, and stable profiling and quantitation of exosomes in real-case biospecimens toward further diagnostics.

### Assay of clinical sample-derived exosome for NSCLC diagnosis

**Fig. 5:**
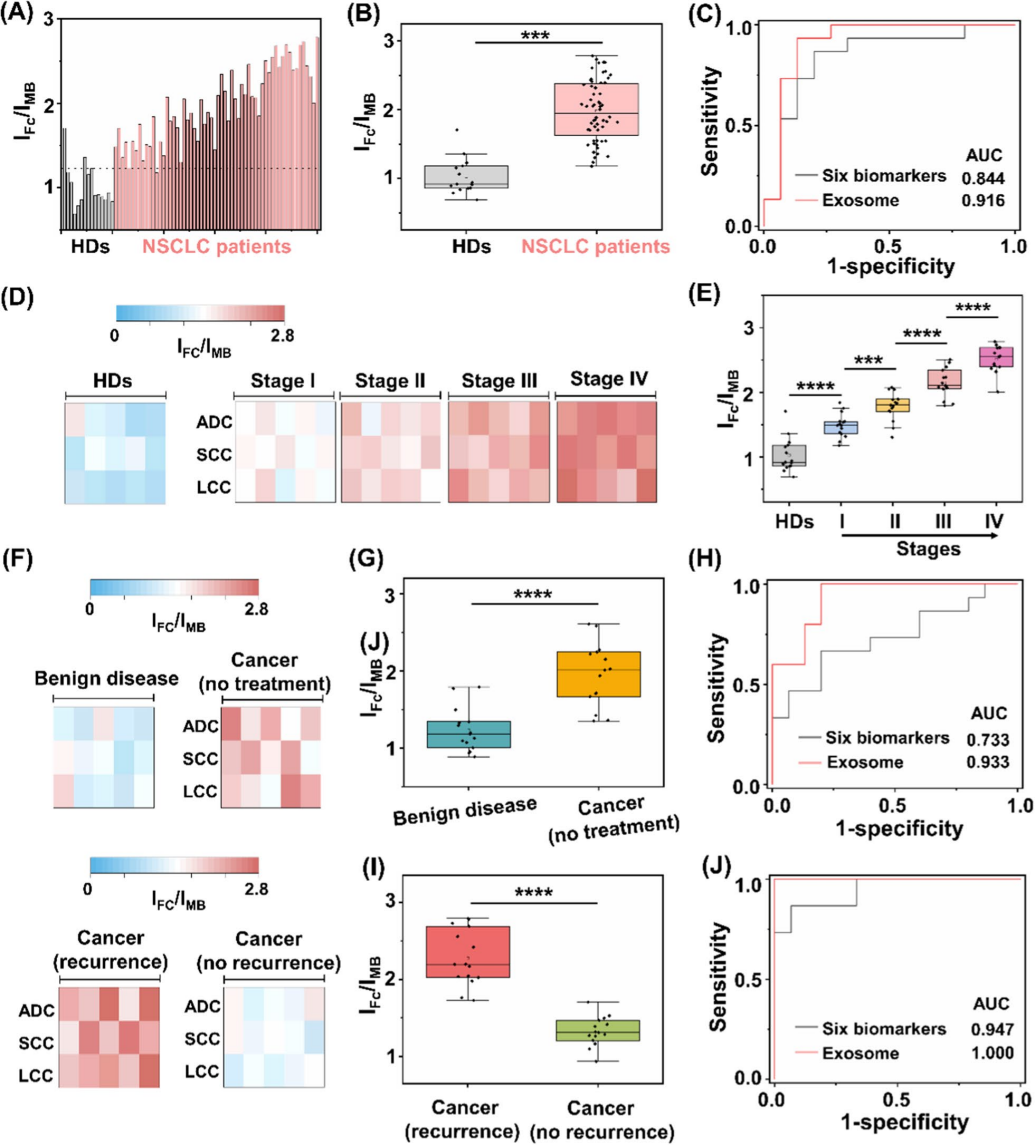
Assay of clinical sample-derived exosome for NSCLC diagnosis. **A** Ratiometric levels and **B** the corresponding scatter intervals of serum samples using the exosome-based assay, to differentiate NSCLC patients from healthy donors (HDs). The optimized threshold (10^7^ particles µL.^−1^) according to the receiver operating characteristic (ROC) curve was denoted by the dashed line in **A**. **C** ROC curves of the exosome-based assay and six clinically adopted serum biomarkers, in differentiating stage I NSCLC patients from HDs. **D** Heat map and **E** scatter intervals of ratiometric intensities for exosome detection in HDs and NSCLC patients at stage I-IV (*n* = 15, respectively). **F** Heat map of ratiometric intensities for exosome detection in patients with benign disease, NSCLC patients without treatment, NSCLC patients with treatment and recurrence/no recurrence (*n* = 15, respectively). **G** Scatter intervals and **H** ROC curves of the exosome-based assay and six clinically adopted serum biomarkers, in differentiating NSCLC patients (stage I, no treatment) from patients with benign disease. **I** Scatter interval for NSCLC patients with treatment and recurrence or not. **J** ROC curves of the exosome-based assay and six clinically adopted serum biomarkers, in differentiating NSCLC patients with recurrence and no recurrence. ***p < 0.001, ****p < 0.0001

Having shown the heterogeneous nature of protein expression on exosome surface, we next demonstrated the possibility of the assay for NSCLC diagnosis. We recruited 60 NSCLC patients with stage I-IV and 15 HDs. All the patient subtypes had been verified by histological findings, including: adenocarcinoma, (ADC, *n* = 20), squamous-cell carcinoma (SCC, *n* = 20), and large-cell carcinoma (LCC, *n* = 20). The sample demographics are provided (Additional file 1: Tables S3–S5). Surface protein markers of serum exosomes were profiled by the assay (Fig. 5A). In particular, marked differences in ratiometric intensity between NSCLC patients and HD group were observed (*p* < 0.001, Fig. 5B). We obtained the optimized diagnostic sensitivity of 98.3% with specificity of 86.7% based on the cut-off value of 1.23 according to the receiver operating characteristic (ROC) curve (area under curve of ROC (AUC) of 0.973 (95%CI: 0.798–1.000)), in discriminating NSCLC patients from HD group. Importantly, the readouts acquired from the assay accounted for a sensitivity of 93.3%, specificity of 86.7% with AUC of 0.916 (95%CI: 0.798–1.000) in the early diagnosis of NSCLC staging I (Fig. 5C).

Exosome overexpression is reported to function as tumor markers, highly correlated with the occurrence and metastasis of NSCLC [66, 67]. We further investigated the applicability of the assay in tumor staging. The ratiometric intensity was strongly predictive of NSCLC progression (Fig. 5D), independent of the cancer subtypes. For instance, early cancer patients (stage I) had higher levels of exosomes as compared to HD, which kept on upregulating in advanced NSCLC (stage IV, *p* < 0.0001). In detail, each stage of NSCLC showed a heterogeneous rise in the exosome level, in contrast with the low expression in HD (*p* < 0.0001, Fig. 5E).

Following cancer treatment, exosomes hold promise as predictive biomarkers for therapy efficacy evaluation and recurrence risk monitoring [68, 69]. We analyzed the exosome protein expression from a new patient cohort (Fig. 5F), including benign disease (*n* = 15); cancer without treatment (*n* = 15); and cancer with treatment (recurrence: *n* = 15; no recurrence: *n* = 15). As depicted in Fig. 5G, NSCLC patients had lower levels of exosome compared to patients with benign disease (t-test, *p* < 0.0001). As a result, we achieved sensitivity of 100.0% and specificity of 80.0% with an AUC of 0.933 (95%CI: 0.847–1.000), in differentiating early NSCLC (stage I, no treatment) from benign disease (red curve in Fig. 5H). We further explored the capability of the assay in predicting the recurrence risk after the surgical/radical operation. We profiled exosome surface proteins and observed significant difference between recurrence and no recurrence groups (t-test, *p* < 0.0001; Fig. 5I). Similarly, we achieved both sensitivity and specificity of 100%, with an AUC of 1.000 (95%CI: 1.000–1.000) in the differentiation of these two groups (red curve in Fig. 5J).

The current liquid biopsy in clinics mainly relies on serum tumor protein markers (*e.g.*, CEA, CA125, SCCA, NSE, Pro-GRP, and CYF21-1) [70–73]. However, the classification models built on the six serum biomarkers underperformed our proposed exosome assay. Consequently, the combination of protein biomarkers demonstrated an AUC of 0.844, 0.733, and 0.947 for the staging, diagnosis, and prognosis of NSCLC in the current datasets (black curves in Fig. 5C, H, J), as well as the reported values (AUC = 0.82, 0.84, and 0.87, respectively) [42, 74]. Importantly, the serum protein markers are far from satisfactory in the early diagnosis of NSCLC (stage I), displaying AUCs ranging 0.460–0.796 consistent with references [23, 75]. Apart from the clinically-adopted serum biomarkers, it was also difficult to differentiate between benign and malignant lesions at an early stage (*e.g.*, SI NSCLC) by other emerging liquid biopsy approaches [72, 76], such as integrated analysis of circulating proteins and mutations in cell-free DNA (CancerSeek) showing an accuracy of 43% and sequencing analysis of circulating tumour DNA (Lung-CLiP) showing a sensitivity of 63% [77, 78]. Taken together, the surface proteins profiled from exosomes by the assay were capable of identifying malignance, superior to the protein biomarkers recommended by medical association guidelines for liquid biopsy of NSCLC.

## Conclusion

As a limitation of our work, larger sample size is needed to further validate the diagnostic performance of the proposed assay. In addition, the integration of the sensing system with microfluidics remains to be a promising path for one-step extraction, separation, and analysis toward the dedicated use of exosome-based assay in clinical settings.

In summary, a sensitive and specific diagnosis assay composed of ratiometric biosensor and OR logic gate was developed for the detection of NSCLC-derived exosomes. The assay enabled ultrasensitive sensing of trace exosomes (as low as 15.1 particles μL^−1^), by combining the HCR amplification with two redox reporter signals. In clinical demonstrations, the assay was superior in detecting stage I NSCLC from HDs with sensitivity of 93.3% than the combination of serum protein biomarkers (AUC of 0.844). In addition, the assay is capable of monitoring the tumor progression, showing an upregulated level of exosome level in advanced NSCLC, in contrast with the low expression in early stage. For patients who underwent biopsies, the readouts acquired from the assay are highly predictive of the cancer recurrence (AUC of 1.000), holding promise in evaluating the treatment efficacy in clinics.

Taken together, the expression of dysregulated surface proteins was profiled and correlated to clinical status during cancer progression. As a proof-of-concept validation, the exosome-based assay provided enhanced differentiation outcomes compared to the reported blood biomarker paradigm for the diagnosis, staging, and prognosis of NSCLC patients. We anticipated the assay could be easily translated into the diagnostic workflow not exclusively specific to malignant tumors.

## Supplementary Information

**Additional file 1: **Materials and reagents, oligonucleotide sequences used in the experiments; GNP synthesis and characterization; Optimization of experimental conditions; Comparison of different methods for exosome detection; Clinical demographics of enrolled cohorts. **Table S1.** Oligonucleotide sequences used in the experiments. **Table S2.** Comparison of different methods for exosome detection. **Table S3.** Clinical demographics for NSCLC diagnosis. **Table S4.** Clinical demographics for differentiation of NSCLC from benign diseases. **Table S5.** Clinical demographics for NSCLC prognosis. **Figure S1.** The intensity of the electrochemical response on (A) the volume ratio of the GNPs and DNA (1 μM), (B) the incubation time between exosomes and aptamers, and (C) the DNA tetrahedron concentration. The concentrations of exosomes were set as 2×10^6^, 6×10^6^, 4×10^6^ particles μL^−1^, respectively. Error bars represented the standard deviation of three repetitive measurements.

## Data Availability

The raw data and processed data required to reproduce these findings are available from the corresponding author upon request.
