# Supplementary material for: Ratiometric electrochemical OR gate assay for NSCLC-derived exosomes
Source: J Nanobiotechnology. 2023 Mar 24;21:104. doi: 10.1186/s12951-023-01833-2 (PMC10037838; doi:10.1186/s12951-023-01833-2)
Supplement: Supplementary file 1 — Additional file 1: Materials and reagents, oligonucleotide sequences used in the experiments; GNP synthesis and characterization; Optimization of experimental conditions; Comparison of different methods for exosome detection; Clinical demographics of enrolled cohorts. Table S1. Oligonucleotide sequences used in the experiments. Table S2. Comparison of different methods for exosome detection. Table S3. Clinical demographics for NSCLC diagnosis. Table S4. Clinical demographics for differentiation of NSCLC from benign diseases. Table S5. Clinical demographics for NSCLC prognosis. Figure S1. The intensity of the electrochemical response on (A) the volume ratio of the GNPs and DNA (1 μM), (B) the incubation time between exosomes and aptamers, and (C) the DNA tetrahedron concentration. The concentrations of exosomes were set as 2×10^6^, 6×10^6^, 4×10^6^ particles μL−1, respectively. Error bars represented the standard deviation of three repetitive measurements. [file 12951_2023_1833_MOESM1_ESM.docx]

Additional Information

**Ratiometric Electrochemical OR Gate Assay for NSCLC-derived Exosomes**

*Fanyu Meng*^1,2 †^*,* *Wenjun Yu*^1†^, *Minjia Niu*^2 †^*,* *Xiaoting Tian^2^, Yayou Miao^2^, Xvelian Li^2^, Yan Zhou^2^, Lifang Ma^1^, Xiao Zhang^2^, Kun Qian^3^, Yongchun Yu^1,2^*, Jiayi Wang^1,2^* and Lin Huang^1,2^**

^1^ Department of Clinical Laboratory Medicine, Shanghai Chest Hospital, Shanghai Jiao Tong University School of Medicine, Shanghai, 200030, China.

^2^ Shanghai Institute of Thoracic Oncology, Shanghai Chest Hospital, Shanghai Jiao Tong University School of Medicine, Shanghai, 200030, China.

^3^ State Key Laboratory for Oncogenes and Related Genes, School of Biomedical Engineering, Institute of Medical Robotics and Med-X Research Institute, Shanghai Jiao Tong University, Shanghai, 200030, P. R. China.

^†^These authors contributed equally.

*Correspondence:

yyc2166@sjtu.edu.cn; jiayi.wang@sjtu.edu.cn; linhuang@shsmu.edu.cn.

**1. Materials and reagents.**

HPLC-purified DNA oligonucleotides (listed in Table S1) and DNA marker were synthesized by Sangon Biotech Co., Ltd. (Shanghai, China). All oligonucleotides were dissolved in 10 mM Tris buffer (pH 7.4) and stored at 4℃ for further use. Mercaptohexanol (98.0%, MCH), Gold (III) chloride trihydrate (HAuCl_4_∙3H_2_O), Tris(2-carboxyethyl)phosphine hydrochloride (TCEP), trisodium citrate, and 4-(2-hydroxyethyl)piperazine-1-ethanesulfonic acid (HEPES) were purchased from Aladdin Reagent Co., Ltd (Shanghai, China). Gel electrophoresis loading buffer was purchased from Solarbio. Co., Ltd. (Shanghai, China), which were of analytical grade and used without further purification or treatment. ExoQuick™ Kits were purchased from System Biosciences (SBI, USA). MiRCURY® Exosome Kits were obtained from Qiagen (Hilden, Germany). Tumor biomarkers were purchased from Tellgen Co. Ltd. (Shanghai, China), including carcinoembryonic antigen, neuronspecificenolase, squamous cell carcinoma antigen, pro-gastrin-releasing peptide, cytokeratin fragment 19, and carbohydrate antigen125. The human lung cancer cell lines were purchased from the Institute of Biochemistry and Cell Biology, Chinese Academy of Sciences (Shanghai, China). Human serum samples were provided by the Shanghai Chest Hospital affiliated to Shanghai Jiao Tong University School of Medicine, under the approved protocol NO. KS22025. Ultrapure water was used (18.2 MΩ cm, Milli-Q, Millipore, GmbH) throughout experiments.

**Table S1.** Oligonucleotide sequences used in the experiments.

|  | Sequence (5'-3') | | |
| --- | --- | --- | --- |
| D1 | | | ACATTCCTAAGTCTGAAACATTACAGCTTGCTACACGAG  AAGAGCCGCCATAGTATTTTTTCATCGAGTT |
| D2 | | | ACATTCCTAAGTCTGAAACATTACAGCTTGCTACACGAG  AAGAGCCGCCATAGTATTTTTTGGTTGCGTC |
| D3 | | | HS-TATCACCAGGCAGTTGACAGTGTAGCAAGCTGTAATAGA  TGCGAGGGTCCAATAC |
| D4 | | | HS-TCAACTGCCTGGTGATAATACGACACTACGTGGGAATCT  ACTATGGCGGCTCTTC |
| D5 | | | HS-TTCAGACTTAGGAATGTCGTTCCCACGTAGTGTCGTTAG  TATTGGACCCTCGCAT |
| Aptamer1 (Apt1) | | | HS-TTTTTTTCGCGCGAGTCGTCTGGGGAACCATCGAGTTAC  ACCGACCTTCTATGTGCGGCCCCCCGCATCGTCCTCCC |
| Aptamer2 (Apt2) | | | HS-TTTTTTCACTACAGAGGTTGCGTCTGTCCCACGTTGTCAT  GGGGGGTTGGCCTG |
| Block strand1 (B1) | | | TCTCTCATACAAACTCGATG |
| Block strand2 (B2) | | | TCTCTCATACAGACGCAACC |
| Substrate strand1 (S1-MB) | | | AACTCGCC-MB |
| Substrate strand2 (S2-MB) | | | GACGCATT-MB |
| Hairpin DNA1 (H1-Fc) | | | TGTATGAGAGATATCATACAGTTTCA-Fc |
| Hairpin DNA1 (H2-Fc) | | | TATCTCTCATACATGAAACTGTATGA-Fc |
| Mismatched 1 | | | HS-TTTTTTTCGCGCGAGTCGTCTGGGGAACCATCGAGTTAC  ACCGACCTTAGCTGTGCGGCCCCCCGCATCGTCCTCCC |
| Mismatched 2 | | | HS-TTTTTTCACTACAGAGGTTGCGTCTGTCCCGAATTGTCAT  GGGGGGTTGGCCTG |
| Control (C) | | HS-TCCTAGCTAGGAGCTGCCCCGAGATCTCTTCTCCGAGC CGGTCGA AATAGT | |

**2. GNP synthesis and characterization.**

GNPs were firstly prepared by using citrate reduction method according to previous reference [1]. In brief, HAuCl_4_ aqueous solution (100 mL, 1 mM) was added into the reaction vessel under constant stirring. Then, 10 mL of sodium citrate (38.8 mM) was quickly added to the boiling solution, and the reaction lasted for 30 minutes until the color changed from light yellow to deep red. The obtained solution was slowly cooled to room temperature and stored at 4℃. The transmission electron microscopy (TEM, HT7800, Japan) images were performed at an acceleration voltage of 200 KV. The ultraviolet-visible (UV-vis) absorption spectra were obtained on a Biotek Epoch2 (America) at room temperature.

**3. Optimization of experimental conditions.**


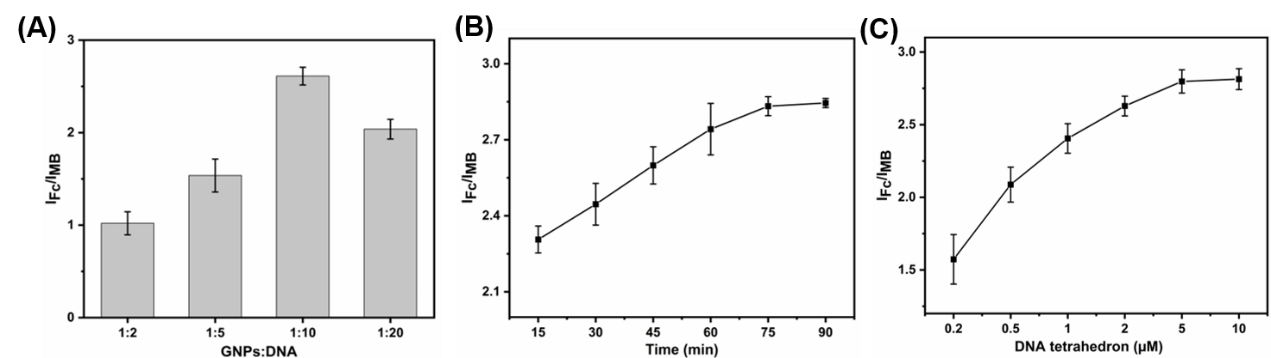


**Fig. S1** The intensity of the electrochemical response on (A) the volume ratio of the GNPs and DNA (1 μM), (B) the incubation time between exosomes and aptamers, and (C) the DNA tetrahedron concentration. The concentrations of exosomes were set as 2×10^6^, 6×10^6^, 4×10^6^ particles μL^−1^, respectively. Error bars represented the standard deviation of three repetitive measurements.

**4. Comparison of different methods for exosome detection.**

Table S2. Comparison of different methods for exosome detection.

| Method | Target | Linear range  (particles μL^−1^) | Detection limit  (particles μL^−1^) | References |
| --- | --- | --- | --- | --- |
| Fluorescence | CD63 | 4.75-47.5 ×10^3^ | ~ 0.00766 | [2] |
| SPR sensor | PD-L1 | 10-5 ×10^3^ | 0.167 | [3] |
| Electrical sensor | CD63 | 1-10^7^ | 100 | [[4](#_ENREF_3)] |
| Electrochemical sensor | ALP/EpCAM | 270-10^5^ | 105 | [[5](#_ENREF_4)] |
| Electrochemical sensor | CD63 | 1-10^5^ | 2.1926 | [[6](#_ENREF_5)] |
| Electrochemical sensor | CEA/EpCAM | 100-10^8^ | 15.1 | This work |

**References**

1. Meng F, Yu W, Chen C, Guo S, Tian X, Miao Y, et al. A versatile electrochemical biosensor for the detection of circulating microrna toward non-small cell lung cancer diagnosis. Small. 2022;18:e2200784.
2. Li S, Zhu L, Zhu L, Mei X, Xu W. A sandwich-based evanescent wave fluorescent biosensor for simple, real-time exosome detection. Biosens Bioelectron. 2022;200:113902.
3. Wang Y, Mao Z, Chen Q, Koh K, Hu X, Chen H. Rapid and sensitive detection of PD-L1 exosomes using Cu-TCPP 2D MOF as a SPR sensitizer. Biosens Bioelectron. 2022;201:113954.
4. Ramadan S, Lobo R, Zhang Y, Xu L, Olena S, Tsang D, et al. Carbon-dot-enhanced graphene field-effect transistors for ultrasensitive detection of exosomes. ACS Appl Mater Inter. 2021;13:7854-64.
5. Moura S, Pallarès-Rusiñol A, Sappia L, Martí M, Pividori M. The activity of alkaline phosphatase in breast cancer exosomes simplifies the biosensing design. Biosens Bioelectron. 2022;198:113826.
6. Lee M, Park S, Kim G, Park C, Lee M, Ahn J, et al. A pretreatment-free electrical capacitance biosensor for exosome detection in undiluted serum. Biosens Bioelectron. 2022;199:113872.

**5. Clinical demographics of enrolled cohorts.**

**Table S3.** Clinical demographics for NSCLC diagnosis.

| Index | Stage | Subtype | CEA  ng/mL | CYF21-1  ng/mL | SCCA  ng/mL | NSE  ng/mL | CA125  U/mL | Pro-GRP  pg/mL |
| --- | --- | --- | --- | --- | --- | --- | --- | --- |
| HD1 | - | - | 1.29 | 1.56 | 0.28 | 13.5 | 10.02 | 45.17 |
| HD2 | - | - | 2.3 | 1.43 | 0.35 | 17.7 | 15.08 | 48.6 |
| HD3 | - | - | 2.74 | 1.85 | 0.28 | 7.81 | 57.27 | 40.47 |
| HD4 | - | - | 1.37 | 1.41 | 0.36 | 11.36 | 12.38 | 50.75 |
| HD5 | - | - | 2.06 | 4.65 | 0.39 | 17.27 | 9.72 | 45.6 |
| HD6 | - | - | 3.66 | 2.22 | 0.76 | 22.31 | 11.2 | 76.4 |
| HD7 | - | - | 4.3 | 3.97 | 0.57 | 15.35 | 18.71 | 68.99 |
| HD8 | - | - | 1.7 | 1.68 | 0.32 | 11.82 | 13.88 | 74.27 |
| HD9 | - | - | 2.08 | 2.22 | 0.42 | 19.48 | 11.49 | 64.61 |
| HD10 | - | - | 2.18 | 2.65 | 0.33 | 8.16 | 7.11 | 49.46 |
| HD11 | - | - | 4.18 | 2.49 | 0.34 | 16.66 | 11.49 | 33.66 |
| HD12 | - | - | 7.14 | 3.74 | 1.69 | 19.29 | 15.38 | 56.79 |
| HD13 | - | - | 0.79 | 1.33 | 0.42 | 17.5 | 7.97 | 40.9 |
| HD14 | - | - | 2.3 | 3.65 | 0.39 | 24.29 | 13.58 | 43.89 |
| HD15 | - | - | 2.27 | 1.59 | 0.28 | 18.85 | 28.26 | 58.09 |
| NSCLC1 | Ⅰ | LA | 417.31 | 7.42 | 0.7 | 12.17 | 50.77 | 70.51 |
| NSCLC2 | Ⅰ | LA | 67.35 | 10.45 | 1.28 | 25.8 | 43.89 | 57.47 |
| NSCLC3 | Ⅰ | LA | 9.28 | 3.94 | 1.83 | 30.09 | 86.83 | 63.4 |
| NSCLC4 | Ⅰ | LA | 4.14 | 2.06 | 0.41 | 5.95 | 20.49 | 36.58 |
| NSCLC5 | Ⅰ | LA | 9.49 | 1.46 | 0.55 | 5.48 | 12.37 | 34.13 |
| NSCLC6 | Ⅰ | SCLC | 2.63 | 2.01 | 1.09 | 16.42 | 12.18 | 66.76 |
| NSCLC7 | Ⅰ | SCLC | 23.92 | 9.91 | 2.24 | 21.88 | 15.16 | 69.76 |
| NSCLC8 | Ⅰ | SCLC | 10.88 | 2.36 | 1.27 | 14.81 | 18.42 | 31.34 |
| NSCLC9 | Ⅰ | SCLC | 1.95 | 1.42 | 0.51 | 8.27 | 19.66 | 35.88 |
| NSCLC10 | Ⅰ | SCLC | 2.68 | 1.33 | 0.78 | 36.02 | 19.24 | 45.11 |
| NSCLC11 | Ⅰ | LCLC | 3.54 | 0.67 | 1.68 | 9.46 | 21.12 | 50.89 |
| NSCLC12 | Ⅰ | LCLC | 3.06 | 3.6 | 0.29 | 28.08 | 9.54 | 55.2 |
| NSCLC13 | Ⅰ | LCLC | 4.72 | 1.8 | 0.64 | 24.93 | 9.82 | 48.31 |
| NSCLC14 | Ⅰ | LCLC | 3.62 | 1.27 | 0.73 | 17.43 | 11.19 | 58.78 |
| NSCLC15 | Ⅰ | LCLC | 2.11 | 1.17 | 1.37 | 16.29 | 7.49 | 54.69 |

**Table S4.** Clinical demographics for differentiation of NSCLC from benign diseases.

| Index | Stage | Subtype | Treatment | CEA  ng/mL | CYF21-1  ng/mL | SCCA  ng/mL | NSE  ng/mL | CA125  U/mL | Pro-GRP  pg/mL |
| --- | --- | --- | --- | --- | --- | --- | --- | --- | --- |
| BD1 | - |  | - | 4.24 | 4.13 | 1.04 | 28.31 | 24.38 | 86.54 |
| BD2 | - |  | - | 2.98 | 8.81 | 0.62 | 2.96 | 61.52 | 23.8 |
| BD3 | - |  | - | 2.06 | 2.73 | 0.39 | 6.03 | 15.97 | 64.89 |
| BD4 | - |  | - | 6.26 | 4.68 | 0.82 | 24.43 | 28.86 | 32.38 |
| BD5 | - |  | - | 2.44 | 2.55 | 1.72 | 27.18 | 15.94 | 95.48 |
| BD6 | - |  | - | 6.2 | 3.43 | 0.21 | 8.89 | 14.36 | 47.27 |
| BD7 | - |  | - | 3.39 | 4.34 | 1.67 | 14.69 | 42.99 | 72.4 |
| BD8 | - |  | - | 4.79 | 2.28 | 0.58 | 21.34 | 20.72 | 63.03 |
| BD9 | - |  | - | 1.11 | 1.39 | 1.07 | 16.45 | 28.13 | 45.11 |
| BD10 | - |  | - | 3.6 | 1.53 | 0.75 | 13.62 | 14.05 | 21.77 |
| BD11 | - |  | - | 1.34 | 1.66 | 1 | 38.49 | 12.57 | 34.48 |
| BD12 | - |  | - | 0.82 | 1.47 | 0.93 | 9.34 | 13.36 | 28.92 |
| BD13 | - |  | - | 1.87 | 1.55 | 0.51 | 12.1 | 30.65 | 53.81 |
| BD14 | - |  | - | 3.75 | 2.36 | 0.7 | 17.25 | 11.53 | 29.96 |
| BD15 | - |  | - | 2.4 | 2.2 | 0.73 | 12.55 | 37.81 | 63.03 |
| NSCLC1 | Ⅰ | LA | NO | 3.78 | 37.7 | 1.55 | 15.89 | 23.46 | 5.87 |
| NSCLC2 | Ⅰ | LA | NO | 5.11 | 28.89 | 0.57 | 19.97 | 28.68 | 3.97 |
| NSCLC3 | Ⅰ | LA | NO | 2.4 | 18.2 | 0.9 | 25.6 | 49.47 | 2.46 |
| NSCLC4 | Ⅰ | LA | NO | 41.93 | 40.25 | 1.33 | 23.39 | 35.61 | 4.79 |
| NSCLC5 | Ⅰ | LA | NO | 26.02 | 36.16 | 1.45 | 12.67 | 30.14 | 1.98 |
| NSCLC6 | Ⅰ | SCLC | NO | 7.39 | 10.13 | 26.32 | 31.9 | 10.9 | 3.11 |
| NSCLC7 | Ⅰ | SCLC | NO | 4.83 | 34.03 | 0.84 | 21.47 | 16.38 | 2.33 |
| NSCLC8 | Ⅰ | SCLC | NO | 2.15 | 24.52 | 0.7 | 24.72 | 72.9 | 6.2 |
| NSCLC9 | Ⅰ | SCLC | NO | 1.75 | 19.01 | 0.62 | 16.5 | 20.3 | 1.44 |
| NSCLC10 | Ⅰ | SCLC | NO | 6.42 | 8.98 | 1.8 | 8.89 | 65.5 | 2.9 |
| NSCLC11 | Ⅰ | LCLC | NO | 0.89 | 7.3 | 1.6 | 18.09 | 7.9 | 2.35 |
| NSCLC12 | Ⅰ | LCLC | NO | 3.29 | 5.28 | 1.0 | 12.01 | 15.0 | 1.52 |
| NSCLC13 | Ⅰ | LCLC | NO | 2.84 | 9.3 | 0.8 | 13.21 | 40.0 | 1.32 |
| NSCLC14 | Ⅰ | LCLC | NO | 5.55 | 15.88 | 5.95 | 16.69 | 26.98 | 7.47 |
| NSCLC15 | Ⅰ | LCLC | NO | 4.4 | 13.71 | 1.2 | 17.82 | 17.65 | 3.63 |

**Table S5.** Clinical demographics for NSCLC prognosis.

| Index | Subtype | Recurrence | CEA  ng/mL | CYF21-1  ng/mL | SCCA  ng/mL | NSE  ng/mL | CA125  U/mL | Pro-GRP  pg/mL |
| --- | --- | --- | --- | --- | --- | --- | --- | --- |
| N1 | LA | NO | 1.21 | 1.09 | 0.28 | 14.04 | 8.39 | 62.43 |
| N2 | LA | NO | 2.88 | 1.78 | 0.3 | 35.44 | 12.92 | 35.62 |
| N3 | LA | NO | 4.28 | 1.78 | 1.77 | 20.52 | 24.76 | 54.54 |
| N4 | LA | NO | 1.27 | 0.75 | 0.45 | 10.16 | 15.46 | 42.5 |
| N5 | LA | NO | 2.13 | 1.12 | 0.28 | 11.05 | 51.13 | 39.22 |
| N6 | SCLC | NO | 3.25 | 1.97 | 0.15 | 16.31 | 33.78 | 39.88 |
| N7 | SCLC | NO | 3.89 | 4.05 | 0.39 | 21.68 | 20.49 | 40.76 |
| N8 | SCLC | NO | 2.94 | 2.44 | 0.08 | 14 | 131.3 | 36.54 |
| N9 | SCLC | NO | 1.83 | 4.22 | 0.71 | 10.76 | 13.19 | 50.6 |
| N10 | SCLC | NO | 3.16 | 1.95 | 1.12 | 19.09 | 9 | 44.63 |
| N11 | LCLC | NO | 3.06 | 3.6 | 0.29 | 28.08 | 9.54 | 55.2 |
| N12 | LCLC | NO | 4.72 | 1.8 | 0.64 | 24.93 | 9.82 | 48.31 |
| N13 | LCLC | NO | 3.62 | 1.27 | 0.73 | 17.43 | 11.19 | 58.78 |
| N14 | LCLC | NO | 4.66 | 5.03 | 0.72 | 23.8 | 17.79 | 35.79 |
| N15 | LCLC | NO | 3.54 | 1.34 | 0.46 | 22.24 | 27.43 | 36.74 |
| N16 | LA | YES | 3.12 | 1.67 | 0.72 | 14.41 | 22.01 | 55.25 |
| N17 | LA | YES | 2.9 | 2.1 | 0.51 | 15.14 | 13.37 | 43.91 |
| N18 | LA | YES | 1.86 | 2.64 | 0.91 | 13.7 | 14.39 | 51.25 |
| N19 | LA | YES | 1.39 | 1.46 | 0.84 | 10.76 | 13.67 | 49.76 |
| N20 | LA | YES | 2.73 | 2.77 | 0.61 | 12.63 | 14.53 | 59.41 |
| N21 | SCLC | YES | 6.57 | 8.09 | 3.73 | 13.69 | 35.46 | 56.93 |
| N22 | SCLC | YES | 2.78 | 3.71 | 0.97 | 8.92 | 85.74 | 34.87 |
| N23 | SCLC | YES | 2.44 | 17.05 | 11.03 | 20.91 | 4.99 | 30.57 |
| N24 | SCLC | YES | 4.56 | 1.38 | 0.45 | 11.43 | 16.21 | 41.03 |
| N25 | SCLC | YES | 9.67 | 6.92 | 1.2 | 18.74 | 13.94 | 49.62 |
| N26 | LCLC | YES | 7.11 | 2.04 | 1.19 | 17.85 | 163.6 | 43.48 |
| N27 | LCLC | YES | 12.43 | 4.97 | 1.13 | 9.03 | 16.21 | 59.62 |
| N28 | LCLC | YES | 14.79 | 1.54 | 0.89 | 16.73 | 18.89 | 50.55 |
| N29 | LCLC | YES | 1.6 | 4.94 | 0.7 | 10.34 | 8.06 | 50.59 |
| N30 | LCLC | YES | 137.5 | 6.23 | 0.75 | 31.29 | 432.21 | 54.69 |
